# Jazia prime vendor system- a public-private partnership to improve medicine availability in Tanzania: from pilot to scale

**DOI:** 10.1186/s40545-019-0163-4

**Published:** 2019-02-25

**Authors:** Karin Wiedenmayer, Romuald Mbwasi, William Mfuko, Ezekiel Mpuya, James Charles, Fiona Chilunda, Denis Mbepera, Ntuli Kapologwe

**Affiliations:** 1 Swiss Tropical and Public Health Institute, Basel, Switzerland; 2 University of Basel, Petersplatz 1, 4051 Basel, Switzerland; 3 Senior pharmaceutical consultant, Dar es Salaam and senior lecturer at St. John’s University of Tanzania, Dodoma, Tanzania; 4 Senior pharmaceutical consultant, Dar es Salaam, Dodoma, Tanzania; 5 Health System Resource Center, President’s Office Regional Administration and Local Government, Dodoma, Tanzania; 6 Regional Medical Officer, Regional Administrative Secretary’s Office, Dodoma, Tanzania; 7 Health Promotion and System Strengthening Project, Dodoma, Tanzania; 8 Regional Pharmacist, Regional Administrative Secretary’s Office, Dodoma, Tanzania; 9 Director of Health Services, Social Welfare and Nutrition Services, President’s Office of Regional Administration and Local Government, Dodoma, Tanzania

**Keywords:** Supply chain, Public-private partnership, Pharmaceutical procurement, Medicines management, Pilot to scale, National roll-out, Prime vendor, Tanzania

## Abstract

**Background:**

The availability of medicines in public health facilities in Tanzania is problematic. Medicines shortages are often caused by unavailability at Medical Stores Department, the national supplier for public health facilities. During such stock-outs, districts may purchase from private suppliers. However, this procedure is intransparent, bureaucratic and uneconomic.

**Objectives:**

To complement the national supply chain in case of stock-outs with a simplified, transparent and efficient procurement procedure based on a public-private partnership approach with a prime vendor at the regional level. To develop a successful pilot of a Prime Vendor system with the potential for national scale-up.

**Methods:**

A public-private partnership was established engaging one private sector pharmaceutical supplier as the Prime Vendor to provide the complementary medicines needed by public health facilities in Tanzania. The Dodoma pilot region endorsed the concept involving the private sector, and procedures to procure complementary supplies from a single vendor in a pooled regional contract were developed. A supplier was tendered and contracted based on Good Procurement Practice. Pilot implementation was guided by Standard Operating Procedures, and closely monitored with performance indicators. A 12-step approach for national implementation was applied including cascade training from national to facility level. Each selected vendor signed a contract with the respective regional authority.

**Results:**

In the pilot region, tracer medicines availability increased from 69% in 2014 to 94% in 2018. Prime vendor supplies are of assured quality and average prices are comparable to prices of Medical Stores Department. Procurement procedures are simplified, shortened, standardized, transparent and well-governed. Procurement capacity was enhanced at all levels of the health system. Proven successful, the Prime Vendor system pilot was rolled-out nationally, on government request, to all 26 regions of mainland Tanzania, covering 185 councils and 5381 health facilities.

**Conclusion:**

The Prime Vendor system complements regular government supply through a regional contract approach. It is anchored in the structures of the regional health administration and in the decentralisation policy of the country. This partnership with the private sector facilitates procurement of additional supplies within a culture of transparency and accountability. Regional leadership, convincing pilot results and policy dialogue have led to national roll-out. Transferring this smaller-scale supply chain intervention to other regions requires country ownership and support for sustainable operations.

## Background

Access to health care is determined by availability of medicines and medical supplies [1]. Availability of quality medicines in the provision of health care service is an integral part of universal health coverage (UHC) [2]. Medicines are essential for health care service delivery [3, 4] and account for a high proportion of the health care budget and of household expenditure [5]. Limited availability of medicines is a common feature in most of the public health facilities in developing countries [2, 6, 7]. Clinicians depend on effective, safe and good quality medicines to provide adequate health care, and patients equate quality of care with the availability of medicines. Furthermore, availability of medicines affects patient trust to the health care providers [8]. Evidence has shown that shortage of medicines influences health care seeking behaviour [9, 10]. If out of stock, patients suffer and lose confidence in health services. Stock-outs in health facilities discourage people from enrolling into health insurance schemes and shape their decisions for membership renewal in the case of enrolled individuals [11, 12].

A range of actors, processes, and information is needed to get health products to people along a supply chain and throughout the complex health care system. Various players must collaborate to ensure effective, reliable, and flexible supply chains that provide equitable access to health services for all people. The private sector can play an important role in strengthening supply chains for health by creating synergies [13]. These public-private partnerships (PPP) however must be based on policy and a mutual understanding of the duties and benefits for both partners, tailoring collaboration to the socioeconomic and political context, and local environments [14–16]. One example is the Prime Vendor (PV) approach where the government relies on a private sector firm to manage the supply of a line of products, and provides services to customers in an assigned area of responsibility. Diverse setups of vendor supply systems are possible to obtain medical supplies for public health facilities from private sector suppliers [17].

Medical Stores Department (MSD) is the backbone for public supply of medicines and other health commodities in Tanzania. But the organisation faces challenges in filling orders of health facilities leading to stock-outs at service delivery points. An increase in demand for health care services and expanding coverage of interventions, delays in disbursement of funding for health commodities, an inadequate governance framework, management challenges such as shortage of staff and operational inefficiencies have resulted in working capital erosion at MSD with an unsustainable level of debt accumulating [18, 19].

Centrally, the Ministry of Health, Community Development, Gender, Elderly and Children (MoHCDGEC) allocates defined sums for medical supplies for each public health facility directly to MSD. Health facilities have three main sources of funding for their supplies: direct funding deposited at MSD by the government, health basket funds^1^ and complementary funds collected by the health facility. These include funds from the community health fund (CHF)^2^, national health insurance, and cost-sharing (user fees). Health facilities dedicate up to 67 % of these complementary funds to the purchase of medicines and health commodities, when not available at MSD.

### Problem statement

A review of the national supply chain in Tanzania in 2013 revealed that despite some gains made by vertical programs, general essential medicines availability remained insufficient, resulting in frequent stock-outs of medicines at health service delivery points [18]. Similarly, in Dodoma region, a comprehensive baseline survey in 2011 revealed an availability of essential medicines of 53% with a corresponding stock-out rate of 47%, based on 24 tracer medicines (unpublished: Swiss TPH. HPSS project, internal report. 2013). The order fulfilment rate by Medical Stores Department was 58.6%.

The supply gap of more than 40% stemming from the out-of-stock situation and low order fulfilment rates for supplies by MSD needs to be complemented through other sources. Previously, health facilities filled this gap with purchases by quotation and using complementary funds and health basket funds from multiple private sources, within and outside Dodoma region, incurring high opportunity costs (travel and fuel, per diems, high prices of medicines) in the process and making the whole task of filling this gap cost inefficient. The procedure was uneconomic, bureaucratic, non-transparent, and lengthy, while supplies were of questionable quality. Alternative strategies were needed to fill the supply gap and to complement the public sector supply system.

### Approach

To resolve this situation, the Dodoma Regional Administration and Local Government (RALG) embarked on a novel process to establish a Prime Vendor system and to engage, on a public-private partnership (PPP) basis [20], one private sector pharmaceutical vendor as the primary supplier for supplementary medicines and medical supplies needed by public health facilities in the region.

In principle, a Prime Vendor system (PVS) was established in Dodoma region serving as a “one stop shop” which was intended to alleviate opportunity costs previously incurred by health facilities when searching for alternative sources of supplies which they could not obtain from MSD. At health facility level, the complementary funds formerly used to purchase from multiple private sources, were now to be used for purchases from only one appointed Prime Vendor (PV).

This paper summarizes the implementation results of the PVS pilot in Dodoma region, Tanzania, and outlines its evolution from a promising concept to a successful pilot, finally culminating in a nationwide scale-up and rollout of this policy-supported national supply chain solution.

## Methods

### Concept and pilot

In 2012, a concept note, backed by existing laws, policies and guidelines, was widely circulated and discussed among stakeholders in Dodoma region. Councils and the region endorsed a PV concept involving the private sector. This was realised following a regional stakeholders meeting which explored various options for filling the supply gap arising from MSD challenges of meeting health facility needs. After detailed analysis of advantages and disadvantages of various options, the meeting adopted a PPP approach based on a pooled regional contract with one vendor but with individual orders by districts from the contracted vendor only. The methodology for implementation of the PV concept followed a sequence of general steps as presented in Table 1.

**Table 1:** General steps for implementation

Step No	Objective	Expected output
1	Baseline data	Quantification of medicines needs is available Current private procurement practices are analysed Financial management of complementary funds, flow, amount and procedures is assessed
2	Advocacy and buy-in	Consent and buy-in is reached by all stakeholders
3	Administrative structures	A PV technical committee, PV board and temporary tender evaluation committee is appointed with TORs A Jazia PVS office is identified, equipped and staffed A Jazia PVS coordinator is appointed and instructed
4	Jazia PVS documents	All documents driving the Jazia PVS are reviewed and approved
5	Vendor Forum	Interested private suppliers are informed a possible PPP and have prequalification documents
6	Prequalification	A selected number of potential suppliers have been prequalified
7	Tender	A final supplier (Prime Vendor) is selected and approved
8	PPP contracting	PPP contract is negotiated between the regional authority and the private supplier
9	SOP and M&E documents	SOPs and a M&E framework is available and approved
10	Launch	The Jazia PVS is officially launched by signing the contract between regional authority and private supplier
11	PVS training	Regional and district stakeholders and health facility staff are competent in SOP of Jazia PVS activities Operations start
12	Follow-up	Backstopping, M&E and medicines and financial audits are conducted. Enablers and obstacles of Jazia PVS are assessed and regional team is supported

**Table 2:** Standard Operating Procedures

SOP #	Prime Vendor Operational Area	Level
1	Determination of quarterly order quantities to be purchased from PV by health facility	Health facility
2	Health facility orders consolidation and forwarding to PV	District headquarter
3	Receiving and inspection of consignments from PV	District headquarter
4	Inspection of supplies from PV	Health facility
5	Funds transfer & payment to PV	Health facility
6	Lines of communication within PV system	Health facility - district- regional PV office - PV

Regional authorities carried out a transparent prequalification and procurement process to select and contract a private supplier acting as the regional PV, based on Good Procurement Practice (GPP). Requirements for a PV were spelled out in detail in the prequalification and tender documents. Criteria included legal status, litigation history, general capacity and in particular supply experience of medical commodities, financial position, personnel and equipment capabilities and other relevant information. Tenderers had to satisfy all relevant licensing and/or registration and tax requirements, submit a Conflict of Interest declaration and tender prices. These requirements are based on the national Public Procurement Act [21]. Prices from the contracted PV were fixed and comparable to MSD catalogue prices. To assure efficacy, safety and quality in accordance with MoHCDGEC and Tanzania Food and Drug Authority (TFDA) standards, PV supplies had to be limited to and comply with the national essential medicines list, be registered and approved.

A regional PVS office was established within the regional administration, represented by a PV coordinator, a dedicated pharmacist and support staff. Mandated administrative structures such as a Technical Committee and a Board were appointed by the regional authorities to closely manage and support the PVS.

Standard Operating Procedures (SOPs) were elaborated to manage and drive operations of the PVS. A comprehensive but user-friendly handbook with SOPs for health facilities and councils was developed, covering six key operational areas as listed in Table 2:

All stakeholders involved in the PVS were trained in the use of SOPs.

A monitoring and evaluation (M&E) handbook was developed describing the framework for monitoring and evaluating the performance of both the system and the PV. This included instructions on monitoring responsibility and frequency, and performance metrics. The responsibility of semi-annual monitoring was given to the PV coordination office, a regional instance. Sources of data used were the baseline survey and M&E reports. Key supply chain indicators were defined to measure system performance of the PVS model. They include medicines availability based on 24 tracer medicines, PV utilization, delivery lead time to health facility, promptness of payment to PV by district and satisfaction of councils with PV services. Additional indicators to monitor the performance of the PV as a supplier were formulated; they cover physical product quality, order fulfilment rate, delivery lead time and general quality of communication regarding PV services. Regional and district stakeholders and health care workers at facility level were oriented and trained on Jazia PVS activities, with a focus on SOPs and M&E procedures.

In September 2014, the pilot PVS was launched in the presence of the Deputy Minister of Health and regional and local government authorities of Dodoma. Subsequently, the PV system was formally registered with the Business Registration and Licensing Agency in Tanzania as Jazia PVS.

**Fig. 1:**
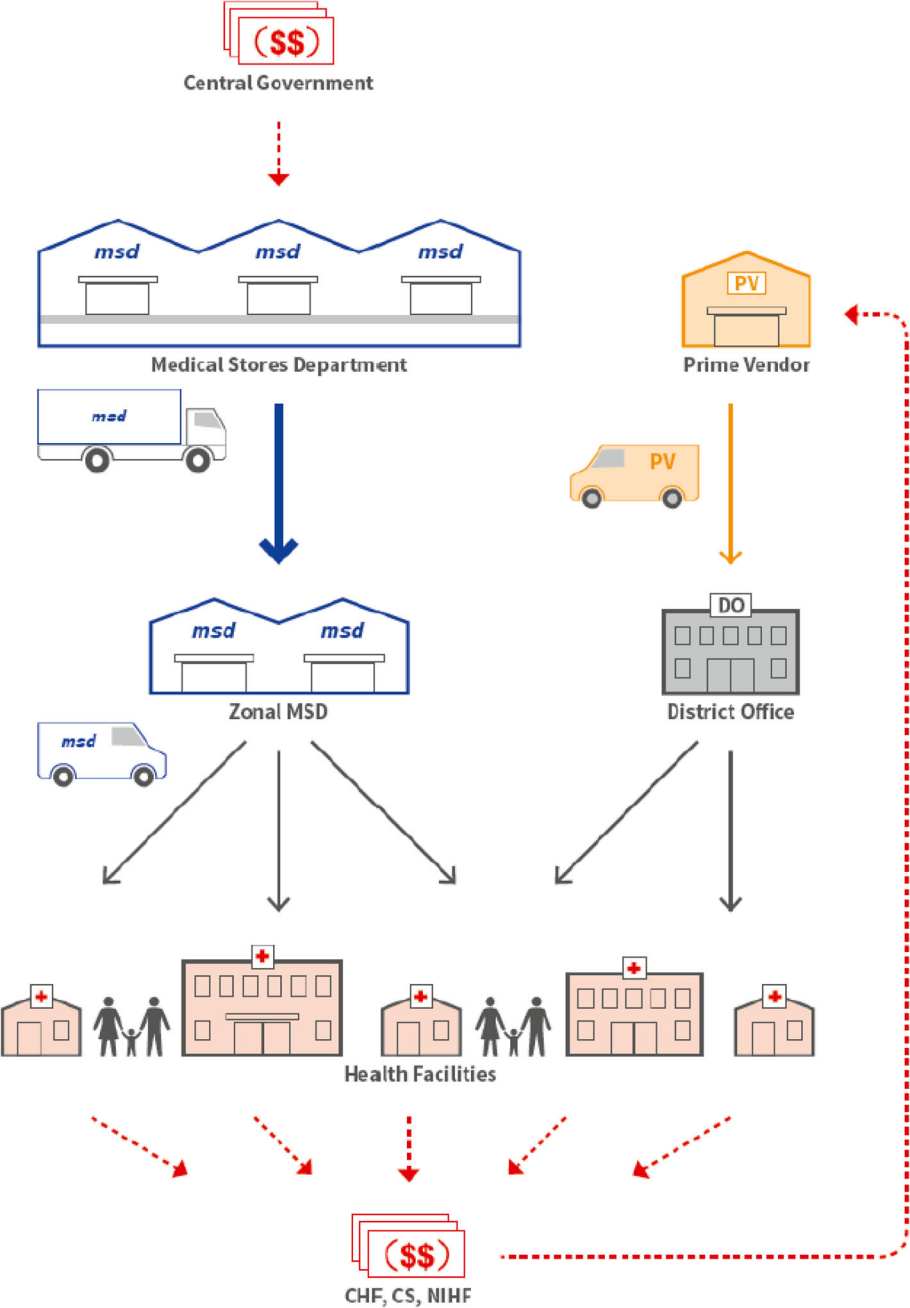
Concept of the fully functioning Jazia Prime Vendor System

The PV concept entailed designing an operational system. Figure 1 below illustrates the fully functional Jazia PVS and the synergy created by the collaboration between MSD and the PVS in improving medicines availability at public health facilities in Dodoma region.

### Accompanying measures and interventions

In addition to the Jazia PVS, a bundle of systemic supply chain interventions was introduced to improve accountability, medicines availability and access to therapy for patients. These include capacity building, peer coaching and auditing. In addition, public financial management was reviewed and revised to standardize and simplify procedures and transactions.

### Scaling up for national roll-out

Following the successful implementation in Dodoma region, the Jazia PVS was expanded to two more regions (Morogoro and Shinyanga) in 2016. This was also stipulated in one of the strategic directions in the Tanzanian Health Sector Strategic Plan IV [22]. In 2017, the Tanzanian government through the President’s Office - Regional Administration and Local Government (PORALG) and the Ministry of Health (MoHCDGEC) requested the national roll-out of the Jazia PV system in all 26 regions of mainland Tanzania. This entailed scaling up to 23 additional regions in Tanzania. The PORALG was able to access co-funding from development partners through the health basket fund. They were integrated in the preparation of the national roll-out plan and participated in the capacity building during cascade training.

A national Coordination Committee was formed, composed of members from ministries and agencies. A task force reviewed documents guiding the establishment of Jazia PVS for adoption. The task included development of a training manual, reviewing Terms of Reference (TOR) for structures supporting the establishment of Jazia PVS and adaptation of tender documents in accordance with the Public Procurement Act guided by the Public Procurement Regulatory Authority.

A comprehensive package of documents including guidelines, TOR, templates and training material was prepared and logistics planned. In principle, the 12-step approach for implementation (see Table 1) was applied.

Training on the Jazia PVS was conducted in a cascaded and stepwise manner. A master training at national level instructing 36 master trainers was followed by training for regional trainers at zonal level. Regional trainers conducted training of district teams in the respective regions. National master trainers were responsible for coordinating and supporting regions to ensure agreed standards. The cascade roll-out ended with the training of health care workers at health facility level. Concurrently, a national Vendor Forum started the tender process. Prequalification of potential vendors and subsequent tendering took place. Each selected vendor (PV) signed a contract with the respective regional authority. After a national launch in October 2018, operations based on the model Jazia PVS of Dodoma region started.

## Results

The establishment of the Jazia PVS fostered an environment of robust and transparent procurement practice, through an innovative public-private partnership. Now, when MSD notifies stock out of items, councils procure complementary medicines individually, using the PV under regional contract, which allows them to benefit from economies of scale. Health facilities manage their own funds, thereby enhancing fiscal decentralization and autonomy. Facility funds are used for pooled purchase from the PV, based on the PPP framework contract. Transparency and accountability in procurement procedures is assured as the PV tender process adheres to the Public Procurement Act. Corruption risks are managed by detailed and strict procurement rules as defined by the Public Procurement Act, a Conflict of Interest declaration, a risk mitigation plan and due diligence procedures.

SOPs guide the process and the purchase of medicines from the PV when these are either out of stock, in short supply or not stocked by MSD. All orders from health facilities are consolidated at the district level and forwarded to the PV. System performance is monitored on semi-annual basis using supply chain metrics defined in a comprehensive M&E framework. Monitoring is conducted by the PV coordination office.

Table 3 summarizes results from Dodoma region, relating the latest monitoring metrics to contractual targets.

**Table 3:** Key performance indicators for Jazia PVS

Indicator for PV System performance	Baseline October 2014	July 2015	July 2016	July 2017	July 2018	Target
Mean availability of tracer medicines (%)	69	75	83	81.5	94	100
PV utilization by health facilities by value of orders (%)	N/A	47	53	50	53	as required
Delivery time from district HQ to health facility (days)	N/A	1–20	14–60	< 5	< 8	< 14
Promptness of payment to PV by district (days)	N/A	14–30	22–300	3–117	12–90	< 22
Councils satisfaction with PV services (score)	N/A	20	17	18	19	20
Indicator for PV (supplier) performance						Target as per PV contract
Overall physical product quality (score)	N/A	20	19	20	20	20
Order fulfillment rate (score)	N/A	20	25	23	25	25
Delivery lead time from PV to district HQ (score)	N/A	14	13	15	15	15
General quality of communication (score)	N/A	5	3	4	5	5

**Fig. 2:**
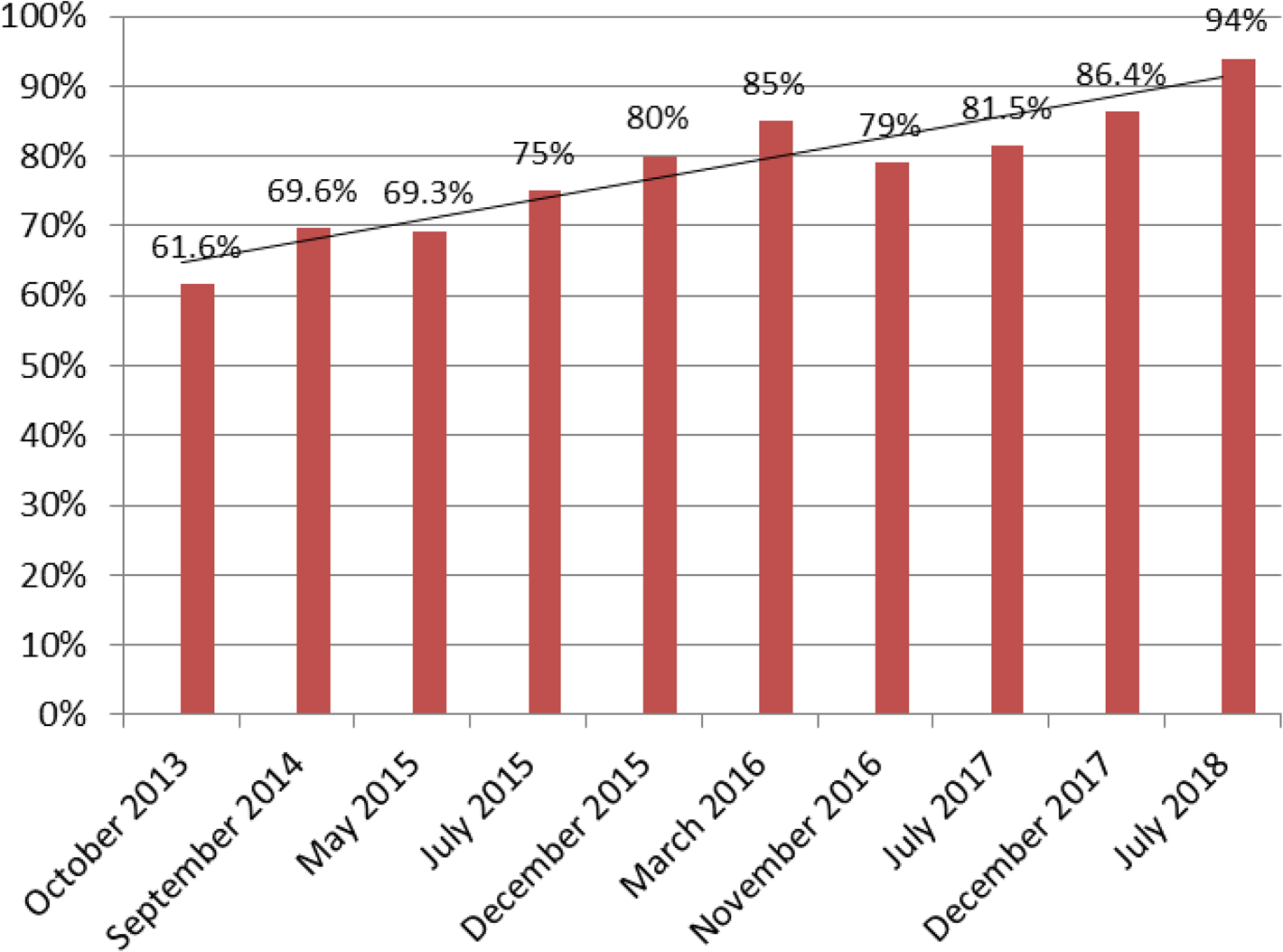
Tracer medicines availability in % (2013–2018), Dodoma region

Tracer medicines availability in the region (mean availability of all districts) increased from 69% in 2014 to 94% in 2018 (Fig. 2). All councils and 31% of health facilities placed orders to the PV by July 2018. Delivery of health commodities from district HQ to the health facilities was done within the contractual delay of 14 days. Satisfaction of councils and health facilities with the PV performance as a complementary supplier was good, as was the overall physical quality of health supplies. Order fulfilment rate of the contracted PV was 99% reaching the maximum score. The PV adhered to and generally significantly beat the contractual delivery time of 22 days with recorded delivery lead times of between 4 and 15 days. General quality of communication and client responsiveness was recorded as good, with no complaints noted. Payment by the districts for their PV consignments however varied among districts and has seen delays of up to 90 days, not adhering to contractual terms.

In 2018 the Jazia PVS was rolled out from the initial three pilot project regions to an additional 23 regions, thus covering all 26 regions of mainland Tanzania, including 185 councils, 5381 Health facilities and a total population of 51,400,000. The roll-out exercise itself enhanced capacity in procurement procedures and Good Procurement Practice at all levels of the health system in both the public and private sector. The scaling up of the Jazia PVS to all regions of mainland Tanzania being recent, results on key performance indicators are not yet available. Monitoring and close follow-up of metrics will be critical.

## Discussion

The procurement procedure for complementary health supplies when MSD notifies stock-outs was simplified and orders are now coordinated in a standardized and well-governed system approach. The Jazia PVS has replaced a lengthy, bureaucratic and intransparent procurement practice with an efficient, formalized, transparent and innovative PPP model. Monitoring of performance indicators has shown important improvement in the supply chain. Mean availability of medicines at health facilities in the pilot region of Dodoma increased by over 35% between 2014 and 2018.

Client responsiveness and the contractual business relationship of the PPP have contributed to this result. Delivery time for complementary supplies is short and order fulfilment rate is high. Only 31% of health facilities placed orders to the PV during the last semi-annual monitoring period. This may indicate stock sufficiency but it could also mean that some facilities are not yet sufficiently familiar with the procedures of the complementary supply system.

A PPP is based on a mutual understanding of the duties and benefits for both partners. In the pilot region, several districts initially delayed payment to the PV. This was due to weak financial management and to resistance to a transparent new supply system. Mitigating measures were simplification of financial transfers, continued persuasion of all actors regarding the successful intervention, pressure and sanctions by local authorities, as well as visibility of good performance in the districts [23]. Delay in payment from public sector side in the pilot regions however is still reported. Another initial challenge was compliance with SOPs at district and health facility level. This has improved after repeated training followed by internal coaching and supervision. A study in Tanzania found that despite supporting policies, there is limited understanding and recognition of the PPP concept at district level, coupled with reluctance to engage with non-state actors and mistrust towards the private sector [24]. Generally, in this specific PPP, the private supplier adhered better to contractual terms than the public sector districts.

Encouraging metrics, convincing and visible results as well as positive perceptions of the Jazia PVS in the pilot regions have facilitated evidence-based policy dialogue. The challenges of MSD struggling with increasing demands and procurement difficulties prompted the Tanzanian government to initiate a national roll-out of the Jazia PVS as a complementary source of medical supplies. The required advocacy and initial development of tools, tendering and establishing a regional PV system was time-intensive and required substantial lobbying and dialogue with stakeholders and decision makers.

Enabling factors for the successful implementation of the pilot included strong political will and support by the regional secretariat and Health Management Team, a deep sense of ownership of Jazia PVS by the region and districts, and an engaged project implementation team. Constructive collaboration with MSD, TFDA and health ministries was instrumental for national dialogue. Particularly facilitating was the sustained leadership by committed district and regional medical officers. More practical instruments and drivers were a government circular to underpin and instruct purchases by health facilities limited to the two approved suppliers (MSD and the PV), incorporation of PV operations into the regional management’s routine operations and recognition of good performance. Participation and engagement of all actors created ownership and pride in the functional system. A systemic approach to supply chain management including a range of accompanying activities in pharmaceutical management and accountability was crucial. These enabling factors were further enhanced by regular meetings with stakeholders, integration of pharmaceutical staff in decision-making and operational research. Dissemination of results and regular policy dialogue contributed to acceptance and ownership.

Could the significant increase in availability of medicines in the pilot region of Dodoma have happened without the Jazia PVS? The health system is a highly complex system with variety of interactions, interdependence of functions, constantly evolving, adapting, permeable and non-linear in its organisation. The supply chain itself is a feedback loop and complex structure with various junctures that affect the final expected outcome of health commodity availability. Therefore, various factors contribute to the success or failure of a supply chain. Nonetheless, the results of the Jazia PVS pilot certainly indicate a plausible correlation.

The Prime Vendor model in medical supply chains is not new. A successful PV system has been implemented for instance under The Pharmaceutical Prime Vendor Program, a contracting arrangement, through which the Department of Veterans Affairs in the USA purchases prescription drugs and medical supplies for outpatients [25].

In Zimbabwe, a vendor system was implemented where high-cost, slow-moving specialty supplies for hospitals were ordered and directly delivered from a government-selected private supplier [26]. In Tanzania, the Mission for Essential Medical Supplies (MEMS) developed a PV system in 2004 using pooled procurement to purchase from a single supplier. However the contracted supplier had difficulties meeting contractual terms, due to underestimating the complexity of the program, and the PVS failed [17]. Another example of a PV is a project funded by the President’s Emergency Plan for AIDS Relief (PEPFAR) which established a “Prime Vendor” in Tanzania, procuring drugs from approved wholesalers at approved prices. Implementing partners place orders directly with the PV [17]. This approach differs from the Jazia PVS in that it does not complement MSD but delivers a separate and uninterrupted supply of high-quality, affordable products. In contrast, the Jazia PVS is based on a PPP with fixed prices comparable to MSD and supplies are funded from health facility own sources, responding to fiscal decentralization. It therefore comprises a system and not just a vendor approach. Further, Jazia PVS is not a parallel system but anchored within regional structures based hence assuring sustainability. Transparency in the procurement procedures has been a key objective.

The Jazia PVS model can be adapted in health sectors of other countries. However, the local context will determine its operational design. The case study in Tanzania that is described here is specific and embedded in the larger health system context, responding to locally articulated needs, available resources, policies and challenges. Most importantly, a systemic approach is required, embedding the PVS in a given health system and its structural, economic and political characteristics.

Scaling up means expansion or replication of an innovative and effective pilot or small-scale projects to reach more people and broaden the effectiveness of the intervention. Promoting and managing a novel system, dissemination and implementation of innovative and effective public health interventions, however, present challenges both for implementers and for the health workforce. Milat et al. [27] reviewed models and success factors for scaling up public health interventions. Promoting factors included among others a robust monitoring and evaluation system, active engagement of implementers and target population with participatory approaches, strong leadership and the use of evidence. Similarly, WHO identifies three fairly robust generalizations and requirements that promise success of scaling up of public health interventions [28]. “*First, a partnership of organizations working on service delivery, financing and stewardship (co-ordination, regulation etc.)”.*This was achieved in in the pilot region Dodoma by intense and ongoing brainstorming, consultations, participatory meetings and policy dialogue at all levels of the health system. “*Second, a highly committed group of individuals to push it along”*. The Jazia PVS project team together with highly engaged regional actors were able to implement and provide convincing evidence to support continuation and expansion of the pilot. Finally, WHO identifies “*monitoring implementation of the scale-up”* as critical for assessing progress relative to overall objectives and for identifying aspects which are not working well. Also relevant and recommended is the input of external experts steering the project and political support at national level. The main hindering factors have been documented as financial issues and the amount of administrative work [29]. Pilot projects tend to be implemented with a level of input and support that subsequently cannot be sustained when innovations are taken to scale [30].

The Tanzanian PVS coordination team is well aware that introducing a model in other quite diverse regions requires intense and close follow-up and support. Here the Tanzanian government is called to support, coordinate and monitor and to react to any challenges that may arise. Transferring successful smaller-scale initiatives such as this supply chain intervention to other regions and to a larger scale requires taking differences between settings into account. Promoting factors include personal commitment of the local actors and recognizable benefit for the population. This has been well achieved in the original pilot regions and now needs to be replicated in all regions of Tanzania. Resistance to change, particularly to increased transparency and governance in the supply chain of valuable health commodities is imminent. Therefore political support at regional level and experience of project partners is essential.

The government is establishing a centralized Jazia PVS coordination office to effectively manage, monitor and backstop the complementary supply system in all 26 regions. Further capacity building and support for the organizational development is required until the Jazia PVS is solidly integrated in regional structures and fully operational in all regions of mainland Tanzania in collaboration with MSD. The government and related ministries will need to capitalize on their now expanded capacity and maintain the technical knowledge gained.

The national roll-out of this successful pilot to strengthen the public supply chain will face challenges and risks. For instance, the pilot regions received close support and monitoring which allowed taking appropriate measures where needed due to external assistance, favourable regional ownership and strong leadership. Other regions will receive less close-up support and successful implementation will depend on local leadership and commitment, and support from by the national coordination office. Accountability continues to be a matter of the utmost importance to a well-functioning public health supply chain. Auditing and strong supportive supervision will be critical.

Since the Jazia PVS is anchored in existing regional and district structures with a centralized coordination office, little additional costs will have to be budgeted and absorbed by regions. Nevertheless, sustainability both in terms of operational and financial viability, adequate staff and technical capability will require political commitment.

## Conclusions

While MSD will remain the backbone for medicines supply, Jazia PVS complements MSD efforts and ensures that health facilities have the medicines and medical supplies to meet the needs of the people. The PPP supplements the regular government supply with additional supplies from a single private vendor in a pooled regional approach. The Jazia PVS is anchored in the structures of each regional health administration and in the decentralisation policy of the country. When quality of health care is improved, the population will be motivated to join the insurance schemes which in turn generate funds to ensure supply of medicines and provision of care. It is a new option in that it empowers public health facilities to purchase supplementary medicines and supplies with their own resources through a shortened, standardized and transparent procedure, thus improving medicine availability without compromising quality or cost.

Scaling of the successful pilot model required solid evidence of favourable results, strong leadership and active engagement of stakeholders and implementers, a systemic approach and highly committed individuals advocating for change. To ensure sustained momentum in this endeavour, it will be critical for the government and related ministries, partners and supply chain actors to continue leveraging this intervention for a strengthened health system. Regional ownership has been instrumental and a cornerstone for the success of Jazia PVS in pilot regions. Similarly, country stewardship of this complementary supply chain will be crucial for sustainable and effective operations at national level for 26 regions.

Following the nationwide roll-out of Jazia PVS, procurement of complementary medical supplies when MSD is out of stock now operates within a culture of transparency and accountability, based on simplified and standardized procedures in partnership with the private sector.

## Abbreviations

CHF: Community Health Fund
DP: Development Partners
GPP: Good Procurement Practice
HPSS: Health Promotion and System Strengthening
MoF: Ministry of Finance
MoHCDGEC: Ministry of Health, Community Development, Gender, Elderly and Children
MSD: Medical Stores Department
PORALG: President’s Office - Regional Administration and Local Government
PPP: Public-Private Partnership
PPRA: Public Procurement Regulatory Authority
PV: Prime Vendor
PVS: Prime Vendor system
RALG: Regional Administration and Local Government
RHMT: Regional Health Management Team
SDC: Swiss Agency for Development and Cooperation
SOP: Standard Operating Procedures
TFDA: Tanzania Food and Drug Authority
TOR: Terms of Reference
TOT: Training of Trainers
UHC: Universal Health Coverage
WHO: World Health Organization

## Footnotes

1 The Health Basket is a funding mechanism initiated in1999 as part of the Government of Tanzania’s decision to pursue a sector-wide approach (SWAp) in the health sector. The basket is funded by a number of Development Agencies that pool un-earmarked resources to support the implementation of the Health Sector Strategic Plan.

2 The Community Health Fund (CHF) was established by the Government of Tanzania with the support of the World Bank. CHFs are a voluntary community-based financing scheme in which households pay contributions to finance part of their basic health care services to complement the government health financing efforts.

## References

[1] Srivastava D, McGuire A (2015). Patient access to health care and medicines across low-income countries. Soc Sci Med.

[2] Prinja S, Bahuguna P, Tripathy JP, Kumar R (2015). Availability of medicines in public sector health facilities of two north Indian states. BMC Pharmacol Toxicol.

[3] Bigdeli M, Laing R, Tomson G, Babar Z (2015). Medicines and universal health coverage: challenges and opportunities. J Pharm Policy Pract.

[4] Obare V, Brolan CE, Hill PS (2014). Indicators for universal health coverage: can Kenya comply with the proposed post-2015 monitoring recommendations?. Int J Equity Health.

[5] Lu Y, Hernandez P, Abegunde D, Edejer T. Medicine expenditures. In: The world medicines situation 2011. World Health Organization. 2011 (http://apps.who.int/medicinedocs/documents/s18767en/s18767en.pdf, Accessed date 14 Oct 2018.

[6] Shayo EH, Senkoro KP, Momburi R, Olsen OE, Byskov J, Makundi EA, Kamuzora P, Mboera LE (2016). Access and utilisation of healthcare services in rural Tanzania: a comparison of public and non-public facilities using quality, equity, and trust dimensions. Glob Public Health.

[7] Ushie BA, Ugal DB, Ingwu JA (2016). Overdependence on for-profit pharmacies: a descriptive survey of user evaluation of medicines availability in public hospitals in selected Nigerian states. PLoS One.

[8] Shan L, Li Y, Ding D, Wu Q, Liu C, Jiao M, Hao Y, Han Y, Gao L, Hao J, Wang L, Xu W, Ren J (2016). Patient satisfaction with hospital inpatient care: effects of trust, medical insurance and perceived quality of care. PLoS One.

[9] Anselmi L, Lagarde M, Hanson K (2015). Health service availability and health seeking behaviour in resource poor settings: evidence from Mozambique. Health Econ Rev.

[10] Mwabu G, Ainsworth M, Nyamete A (1993). Quality of medical care and choice of medical treatment in Kenya: an empirical analysis. J Hum Resour.

[11] Kalolo A., Radermacher R, Stoermer M, Meshack M, De Allegri M. Factors affecting adoption, implementation fidelity, and sustainability of the redesigned community health Fund in Tanzania: a mixed methods protocol for process evaluation in the Dodoma region. Glob Health Action 2015; 8: 10.3402. 10.3402/gha.v8.29648 PMC4683988 26679408

[12] Agyepong IA, Abankwah DNY, Abroso A, Chun C, Dodoo JNO, Lee S, Mensah SA, Musah M, Twum A, Oh J, Park J, Yang D, Yoon K, Otoo N, Asenso-Boadi F (2016). The "Universal" in UHC and Ghana's National Health Insurance Scheme: policy and implementation challenges and dilemmas of a lower middle income country. BMC Health Serv Res.

[13] White J, O’Hanlon B, Chee G, Malangalila E, Kimambo A, Coarasa J, Callahan S, Levey IR, McKeon K (2013). Tanzania private sector assessment. Bethesda, MD: strengthening health outcomes through the private sector project, Abt Associates Inc.

[14] Reich M (2002). Public-private partnership for public health. Harvard Center for Population and Development Studies.

[15] Itika JS, Mashindano O, Kessy F (2011). Success and Constraints for Improving Public Private Partnership in Health Services Delivery in Tanzania. Dar es Salaam: The Economic and Social Research Foundation (ESRF).

[16] John Snow, Inc. Getting products to people, How Private Sector Solutions Can Strengthen Supply Chains for Public Health, Arlington, VA: John Snow, Inc. 2016.

[17] Management Sciences for Health. MDS-3: Managing access to medicines and health technologies. Arlington: Management Sciences for Health; 2012. (https://www.msh.org/), Accessed date 14 Oct 2018

[18] Printz N, Amenyah J, Serumaga B, Van Wyk D (2013). Tanzania: Strategic review of the national supply chain for health commodities, SCMS and DELIVER project.

[19] Deloitte Consulting Ltd. Strategic review of the Medical Stores Department of Tanzania. The journey to efficiency. Final report. Dar es Salaam: Deloitte Consulting Limited; 2015.

[20] United Republic of Tanzania, Prime Minister’s Office (2009). National public private partnership (PPP) policy.

[21] United Republic of Tanzania (2011). The Public Procurement Act.

[22] United Republic of Tanzania, Ministry of Health and Social Welfare. Health Sector Strategic Plan July 2015 – June 2020 (HSSP IV). 2015. Accessed date 14 Jun 2018

[23] Kapologwe N, Mori AT, Chilunda F, Meshack M, Kalolo A, Wiedenmayer K. Reforming supportive supervision of medicines management with an audit tool in primary health care facilities: a case study of Bahi district. Tanzania Int J Pharm. 2014;4:108–14. Accessed date 14 Jun 2018

[24] Kamugumya D, Olivier J. Health system's barriers hindering implementation of public-private partnership at the district level: a case study of partnership for improved reproductive and child health services provision in Tanzania. BMC Health Serv Res. 2016;16(1):596. Accessed date 14 Jun 2018 10.1186/s12913-016-1831-6 PMC5073970 27769234

[25] Gansler J, Lucyshyn W, Harrington L, Cotton Corl A. Prime Vendor Contracting: Lessons Learned. Center for Public Policy and Private Enterprise. School of Public Policy, University of Maryland. March 2011. Accessed date 14 Jun 2018

[26] Center for Pharmaceutical Management. Access to essential medicines: Tanzania, 2001. Prepared for the strategies for enhancing access to medicines program. Arlington: Management Sciences for Health; 2003. Accessed date 14 Jun 2018

[27] Milat AJ, Bauman A, Redman S. Narrative review of models and success factors for scaling up public health interventions. Implement Sci. 2015;10:113. Accessed date 14 Jun 2018 10.1186/s13012-015-0301-6 PMC4533941 26264351

[28] World Health Organisation. Scaling up Health Services: Challenges and Choices. Technical Brief No.3. 2008. Accessed date 14 Jun 2018

[29] World Health Organization. Scaling up projects and initiatives for better health: from concepts to practice. 2016. (http://www.euro.who.int/en/publications/abstracts/scaling-up-projects-and-initiatives-for-better-health-from-concepts-to-practice-2016, Accessed date 14 Jun 2018

[30] ExpandNet, World Health Organization. Beginning with the end in mind. Planning pilot projects and other programmatic research for successful scaling up. Geneva, World Health Organization. 2011. Accessed date 14 Jun 2018

